# Acidic dileucine motifs in the cylindrical inclusion protein of turnip mosaic virus are crucial for endosomal targeting and viral replication

**DOI:** 10.1111/mpp.13231

**Published:** 2022-05-25

**Authors:** Guanwei Wu, Zhaoxing Jia, Penghuan Rui, Hongying Zheng, Yuwen Lu, Lin Lin, Jiejun Peng, Shaofei Rao, Aiming Wang, Jianping Chen, Fei Yan

**Affiliations:** ^1^ State Key Laboratory for Managing Biotic and Chemical Threats to the Quality and Safety of Agro‐Products, Institute of Plant Virology Ningbo University Ningbo China; ^2^ College of Plant Protection Nanjing Agricultural University Nanjing China; ^3^ London Research and Development Centre Agriculture and Agri‐Food Canada Ottawa Ontario Canada

**Keywords:** acidic dileucine motif, adaptor protein complex 2, cylindrical inclusion protein, endosomes, *Nicotiana benthamiana*, turnip mosaic virus, viral replication

## Abstract

Previously we reported that the multifunctional cylindrical inclusion (CI) protein of turnip mosaic virus (TuMV) is targeted to endosomes through the interaction with the medium subunit of adaptor protein complex 2 (AP2β), which is essential for viral infection. Although several functionally important regions in the CI have been identified, little is known about the determinant(s) for endosomal trafficking. The CI protein contains seven conserved acidic dileucine motifs [(D/E)XXXL(L/I)] typical of endocytic sorting signals recognized by AP2β. Here, we selected five motifs for further study and identified that they all were located in the regions of CI interacting with AP2β. Coimmunoprecipitation assays revealed that alanine substitutions in the each of these acidic dileucine motifs decreased binding with AP2β. Moreover, these CI mutants also showed decreased accumulation of punctate bodies, which enter endocytic‐tracking styryl‐stained endosomes. The mutations were then introduced into a full‐length infectious clone of TuMV, and each mutant had reduced viral replication and systemic infection. The data suggest that the acidic dileucine motifs in CI are indispensable for interacting with AP2β for efficient viral replication. This study provides new insights into the role of endocytic sorting motifs in the intracellular movement of viral proteins for replication.

## Graphical abstract

The acidic dileucine motif (D/E)XXXL(L/I) in the cylindrical inclusion (CI) protein of turnip mosaic virus is recognized by the medium subunit of adaptor complex 2 (AP2β) for endosomal targeting of CI and viral replication.

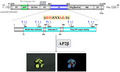

Intracellular membrane traffic relies, to a large extent, on the interactions between adaptor protein (AP) complexes (AP1 to AP5) and the transmembrane cargoes (Owen et al., 2004). AP complexes orchestrate the formation of vesicles destined for transport by distinct intracellular pathways (Park & Guo, 2014). AP complex 2 (AP2) sorts in the endocytic pathway, while AP1 and AP4 facilitate sorting in post‐Golgi compartments. Recognition of either tyrosine‐based (YXXΦ) or dileucine‐based [(D/E)XXXL(L/I)] motifs within the cargo protein by subunits of the AP complex mediates these interactions (X is any amino acid and Φ is a bulky hydrophobic amino acid) (Owen et al., 2004). The AP2 complex is a heterotetramer involved in clathrin‐mediated endocytosis of cargo proteins from the plasma membrane in animal cells. In plants, the AP2 complex is a pentamer consisting of two large (α), one medium (β), and two small (σ and μ) subunits (Fan et al., 2015). The AP2 complex recognizes both the YXXΦ and (D/E)XXXL(L/I) motifs for endocytosis (Bonifacino & Traub, 2003). Dileucine‐based sorting motifs often harbour an acidic residue D/E at position −9, −5, −4, −3 or −2 from the first leucine pair (Lebrun et al., 2018; Park & Guo, 2014; Xiao et al., 2018). The acidic residue D/E is not as crucial as the LL residues for binding to AP2 (Kelly et al., 2008). AP2 complexes play important roles in floral organ development and plant reproduction (Yamaoka et al., 2013). The YXXΦ motif present in the cargo proteins is involved in effector‐triggered immunity (Geldner & Robatzek, 2008; Hatsugai et al., 2016), and little is known about the biological relevance of the interaction of AP2 and the (D/E)XXXL(L/I) motif in cargo proteins.

In addition, the AP complexes have also been implicated in viral infections, particularly human and animal viral infections (Strazic Geljic et al., 2021). Very few studies have been devoted to unravel the involvement of AP2 in plant viral infections. In the case of the plant DNA virus cauliflower mosaic virus (CaMV), it has been reported that its movement protein (MP) contains three YXXΦ motifs that interact with AP2μ, and at least one of these motifs is essential for the localization of MP to endosomes, for tubule assembly, and for viral infection (Carluccio et al., 2014). More recently, we have shown that the medium unit of AP2 (AP2β) from *Arabidopsis* recognizes the replication proteins of turnip mosaic virus (TuMV), a plant RNA virus, as cargoes for endocytosis, endosomal trafficking, and viral infection (Wu et al., 2020).

TuMV is one of the most prevalent pathogens worldwide, causing major losses in economically important vegetable, oilseed, biofuel, forage, and ornamental crops, particularly in the family Brassicaceae (Yang et al., 2021). *Turnip mosaic virus* belongs to the genus *Potyvirus*, and has a single‐stranded, positive‐sense, (ss [+]) RNA genome of c.10 kb that contains a long open reading frame (ORF) and a short ORF resulting from RNA polymerase slippage in the P3 coding sequence (Olspert et al., 2015; Wylie et al., 2017). On translation, the two polyproteins are processed by three viral proteases into 11 mature proteins. Among these, the cylindrical inclusion (CI) protein is a multipartner and multifunctional protein, which has ATPase and RNA helicase activities, and participates in viral genome replication and viral intercellular movement (Revers & Garcia, 2015; Sorel et al., 2014). In a recent study, we reported that AP2β is essential for TuMV replication and mediates the trafficking of TuMV CI in endosomes (Wu et al., 2020). This study was directed to further understand the mechanism underlying the TuMV CI and AP2β interaction for viral endosome trafficking and replication.

We hypothesized that TuMV proteins might bind to AP2 to mediate intracellular traffic for viral replication and therefore we first aimed to identify the region(s) in CI involved in AP2β binding. It is well known that proteins that are intrinsically disordered can obtain the required structure when they bind to their interactors (Charon et al., 2016; Dyson & Wright, 2005). Therefore, the disordered region(s) of the CI were predicted using the PONDR (Predictor of Natural Disordered Regions) website (Figure 1a). The output of three PONDR algorithms predicted four disordered regions: amino acid residues 1–100, residues 101–300, residues 301–500, and residues 501–644. CI was then split accordingly into these four regions (Figure 1b) using the primers listed in Table S1. A bimolecular fluorescence complementation (BiFC) assay was performed in *Nicotiana benthamiana* leaves by agroinfiltration to confirm the interaction of each of these four regions of CI with AtAP2β (see File S1 for experimental procedures). AtAP2β and CI fragments were fused to the C‐terminus of either the N‐proximal or C‐proximal region of the yellow fluorescent protein (YFP) (Tian et al., 2011), respectively. Consistent with our recent report (Wu et al., 2020), there was a positive interaction between full‐length CI and AtAP2β, which occurred in the cytoplasm, showing as punctate bodies around the plasma membrane (PM) (Figure 1c). All four CI fragments also interacted with AtAP2β. CI^101–300^ and CI^501–644^ bound with AtAP2β mainly at the PM, while CI^1–100^ interacted at several punctate bodies in the cytoplasm and the CI^301–500^–AtAP2β complex formed several ring‐like structures in the cytoplasm (Figure 1c; inset). No interaction signals were observed in the negative controls (Figure S1).

**FIGURE 1:**
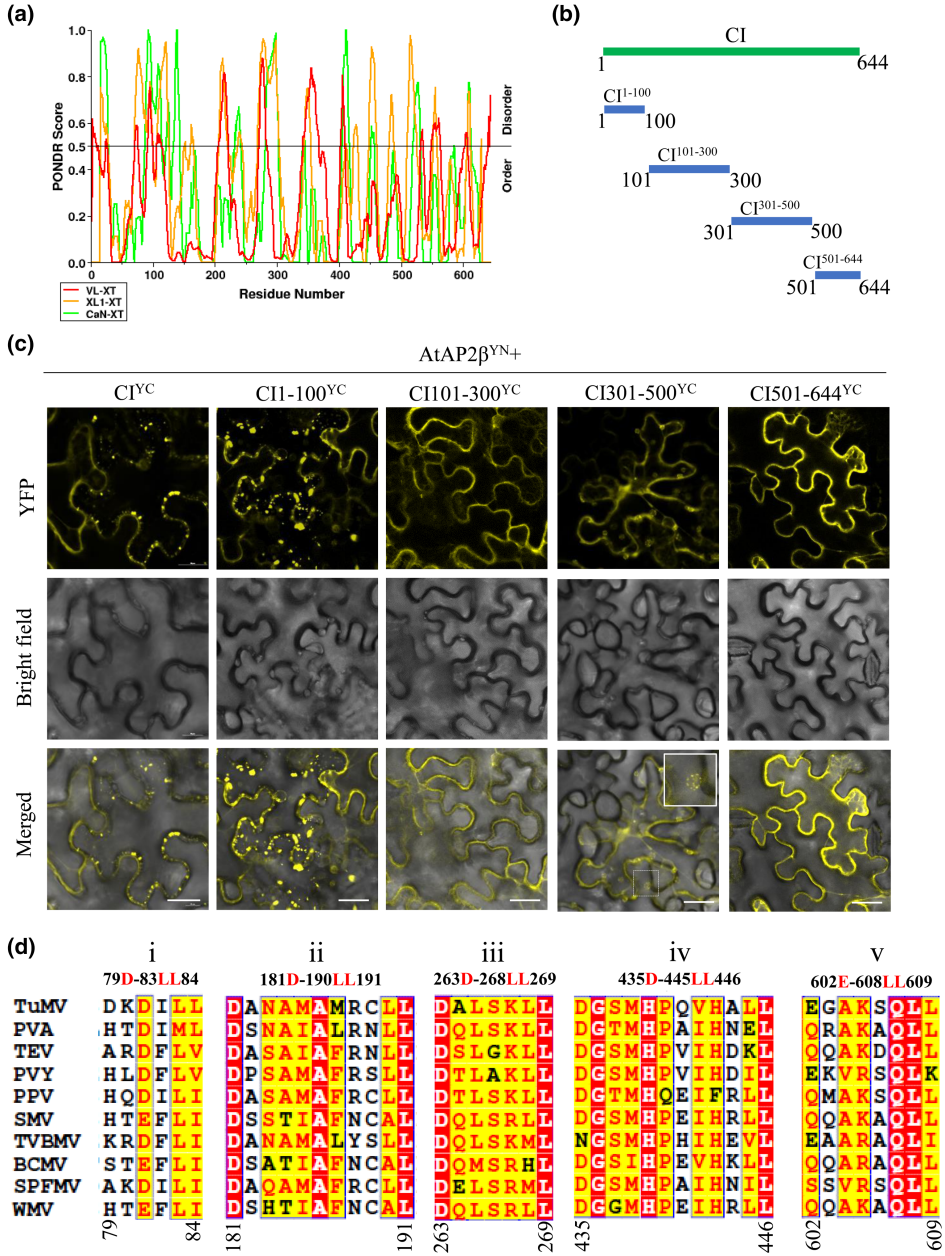
Mapping of the regions in cylindrical inclusion (CI) protein involved in interacting with AtAP2β. (a) Graphical illustration of the intrinsic disorder characteristics of TuMV CI protein. PONDR VL‐XT (red), PONDR XL1‐XT (orange), and PONDR CaN‐XT (green) algorithms were used to analyse the CI protein amino acid sequence. Long solid black lines represent the mean disorder. (b) Schematic representation of the four fragments of the CI protein. (c) Protein–protein interactions between each region and TuMV CI examined by bimolecular fluorescence complementation. Reconstructed yellow fluorescent protein (YFP) signals were observed at 2 days post‐agroinfiltration (dpai). The inset shows a representative ring‐like structure found with CI^301–500^. Scale bar represents 20 μm. (d) Multiple‐sequence alignments of the acidic dileucine motifs in CI protein sequences of 10 potyvirus members. The CI protein sequences of different potyviruses were retrieved from the NCBI GenBank database with the following GenBank accession numbers: turnip mosaic virus (TuMV; EF028235.1), potato virus A (PVA; AJ131402.1), tobacco etch virus (TEV; NC_001555.1), potato virus Y (PVY; AJ439544.2), plum pox virus (PPV; NC_001445.1), soybean mosaic virus (SMV; AF241739.1), tobacco vein banding mosaic virus (TVBMV; EU734432.1), bean common mosaic virus (BCMV; HQ229995.1), sweet potato feathery mottle virus (SPFMV; AB465608.1), and watermelon mosaic virus (WMV; EU660581.1).

The endocytosis inhibitor tyrphostin A23, which targets the YXXΦ motif in cargo proteins (Banbury et al., 2003), has no obvious effect on TuMV infection (Agbeci et al., 2013; Wu et al., 2018). We therefore focused on another endocytic motif (D/E)XXXL(L/I). Inspection of the primary amino acid sequence of TuMV CI revealed seven classical (D/E)XXXL(L/I) sorting motifs in which an acidic residue D or E is located at the −2 to −10 position relative to the first leucine pair or LL residues: 79D‐83LL84, 81D‐83LL84, 181D‐190LL191, 263D‐268LL269, 435D‐445LL446, 550D‐552LI553, and 602E‐608LL609 (Figure S2). Here, we mainly focused on the acidic D/E and LL residues of the CI protein in TuMV infection. The D81 residue in the CI protein has been identified to be important for interaction with the coat protein (CP) and TuMV intercellular movement previously (Deng et al., 2015), thus the 81D‐83LL84 motif was not included here. Five (D/E)XXXLL sorting motifs (79D‐83LL84, 181D‐190LL191, 263D‐268LL269, 435D‐445LL446, and 602E‐608LL609, which we named i to v) were selected for further study. Notably, these five motifs are located in the four candidate binding regions (Figures 1c and S2). Multiple alignments of the CI amino acid sequences among potyviruses indicated that these motifs are conserved among multiple potyviruses (Figure 1d), which could indicate that they play a relevant and conserved role, potentially involved in the interaction with AtAP2β. These results are therefore consistent with the hypothesis that acidic dileucine motifs in the four disordered regions of the CI protein are involved in binding with AtAP2β.

To further dissect the role of these acidic dileucine motifs in binding with AtAP2β, we generated a double mutant (DM) with D/E‐LL to D/E‐AA substitution and a triple mutant (TM) with D/E‐LL to A‐AA substitution for each of the motifs, as shown in Table 1. All 10 mutants interacted with AtAP2β in the BiFC assay and their interaction complexes mainly accumulated along the PM of *N*. *benthamiana* cells (Figure 2a), except for i‐DM, ii‐DM, ii‐TM, and v‐TM. BiFC signals resulting from i‐DM and AtAP2β showed several punctate bodies close to the PM, similar to those seen in interactions between AtAP2β and wild‐type CI or the CI^1–100^ fragment (Figure 1c). The interaction complex between AtAP2β and each of these three mutants (ii‐DM, ii‐TM, and v‐TM) showed several aggregates in the cytoplasm and very weak signals on the PM (Figure 2a). Interestingly, iv‐DM and iv‐TM both interacted with AtAP2β and formed some ring‐like structures, similar to those seen in interactions between AtAP2β and the CI^301–500^ fragment (Figure 1c; inset).

**TABLE 1:** Acidic dileucine motifs in the TuMV cylindrical inclusion (CI) protein and the cloned mutations

Motif Name	Residue positions^a^														
	79	83	84	181	190	191	263	268	269	435	445	446	602	608	609
CI	D – –	L	L	D – –	L	L	D – –	L	L	D – –	L	L	E – –	L	L
i‐DM	D – –	A	A	D – –	L	L	D – –	L	L	D – –	L	L	E – –	L	L
i‐TM	A – –	A	A	D – –	L	L	D – –	L	L	D – –	L	L	E – –	L	L
ii‐DM	D – –	L	L	D – –	A	A	D – –	L	L	D – –	L	L	E – –	L	L
ii‐TM	D – –	L	L	A – –	A	A	D – –	L	L	D – –	L	L	E – –	L	L
iii‐DM	D – –	L	L	D – –	L	L	D – –	A	A	D – –	L	L	E – –	L	L
iii‐TM	D – –	L	L	D – –	L	L	A – –	A	A	D – –	L	L	E – –	L	L
iv‐DM	D – –	L	L	D – –	L	L	D – –	L	L	D – –	A	A	E – –	L	L
iv‐TM	D – –	L	L	D – –	L	L	D – –	L	L	A – –	A	A	E – –	L	L
v‐DM	D – –	L	L	D – –	L	L	D – –	L	L	D – –	L	L	E – –	A	A
v‐TM	D – –	L	L	D – –	L	L	D – –	L	L	D – –	L	L	A – –	A	A

^a^ Residue positions are defined based on the amino acid sequences of TuMV CI (GenBank accession number: ABK27329.1).

**FIGURE 2:**
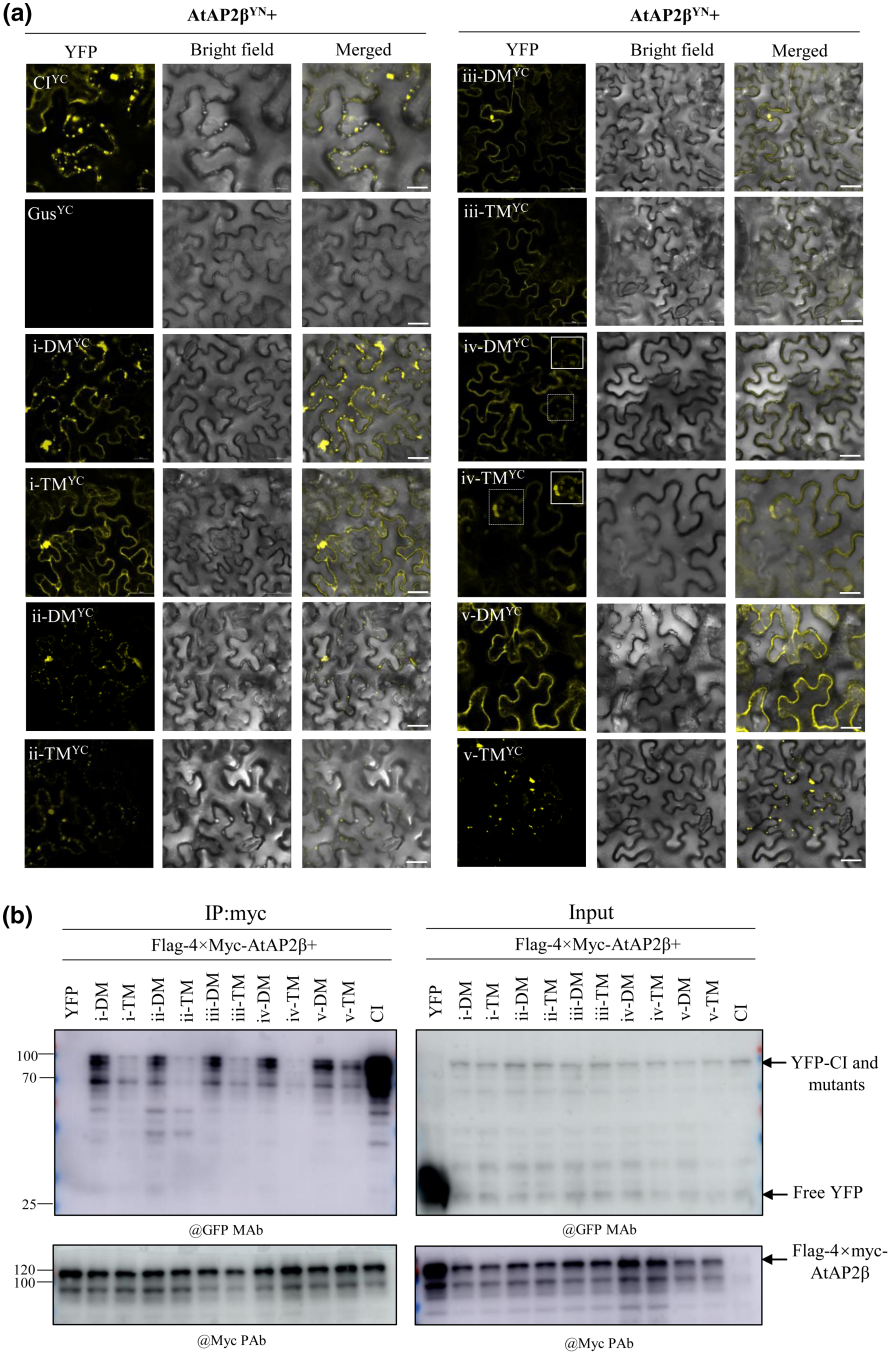
Protein–protein interaction assay between AtAP2β and each of the acidic dileucine motif mutants of cylindrical inclusion (CI) protein. (a) Protein–protein interactions between each CI mutant and AtAP2β in *Nicotiana benthamiana* leaves as shown by bimolecular fluorescence complementation. The inset shows a representative ring‐like structure found with iv‐DM (double mutant) or iv‐TM (triple mutant). Scale bar represents 20 μm. (b) Coimmunoprecipitation assay of protein–protein interactions between each CI mutant and AtAP2β in *N*. *benthamiana* leaves. Different crude plant extracts were immunoprecipitated with anti‐Myc magnetic beads, separated by SDS‐PAGE and immunoblotted with anti‐c‐Myc polyclonal antibody (@Myc PAb) or anti‐GFP monoclonal antibody (@GFP MAb).

To confirm these protein–protein interactions, we conducted a coimmunoprecipitation (co‐IP) assay as previously described (Win et al., 2011; Wu et al., 2022). AtAP2β was cloned into pBA‐FLAG‐4 × Myc‐DC vector (Zhu et al., 2011) to yield the FLAG‐4 × Myc‐tagged construct, and wild‐type CI and its mutants were tagged by an N‐terminal YFP tag. These fusions were transiently coexpressed in *N*. *benthamiana* leaves, followed by co‐IP. As shown in Figure 2b, and consistent with our previous report, the CI protein could be immunoprecipitated with AtAP2β. All 10 CI mutants were able to interact with AtAP2β, but the interaction was weaker than with wild‐type CI, especially for the five triple mutants. Taken together, these data suggest that the absence of just one of the motifs significantly weakens the protein–protein interaction between CI and AtAP2β. The protein mapping and mutagenesis analysis revealed that alanine substitutions in the dileucine motifs altered the intracellular distribution of the CI protein when interacting with AtAP2β (Figures 1 and 2). A key question emerging from this observation is whether the acidic dileucine motifs in TuMV CI are functional internalization motifs. To this end, we investigated the subcellular localization of these CI mutants. Wild‐type CI protein and its mutants fused to an N‐terminal YFP tag were transiently expressed in *N*. *benthamiana* leaves. Confocal microscopy showed that YFP‐CI and all mutants showed similar subcellular localizations, including cytoplasm and nucleus. Interestingly, v‐TM also showed reticular structures in the cytoplasm. Moreover, CI protein accumulated in punctate bodies in the cytoplasm (Figure 3a) and those structures were significantly fewer for the mutants, especially v‐TM (Figure 3b). The punctate bodies of wild‐type CI and its mutants were similar in size (Figure S3). We further colocalized YFP‐CI with N‐(3‐triethylammoniumpropyl)‐4‐(6‐(4‐[diethylamino] phenyl) hexatrienyl) pyridinium dibromide (FM4‐64), an endocytic‐tracking styryl dye used to monitor PM and endosome localization (Bolte et al., 2004b). As shown in Figure 3c, CI‐induced punctate bodies entered FM4‐64‐labelled single membrane vesicle‐like structures, which have been shown to be multivesicular bodies or late endosomes (Bolte et al., 2004a; Cui et al., 2016). Immunoblotting analysis confirmed that wild‐type CI and its mutants all accumulated in *N*. *benthamiana* leaves at the expected protein size (Figure 3d). Replacing both the LL and acidic amino acid (D/E) residues with alanine in each motif had a similar effect to the LL residues substitutions, except for v‐TM, suggesting a critical role of the LL residues in CI internalization for most of CI acidic dileucine motifs. In mammals and yeast, LL residues are recognized by APs 1–3 with a combination of two subunits: AP1 γ‐σ1, AP2 α‐σ2, and AP3 δ‐σ3 hemicomplexes (Doray et al., 2007; Janvier et al., 2003). The binding specificity of plant AP complexes has yet to be elucidated. The acidic dileucine motif of *Arabidopsis* tonoplast‐localized ion transporter VTI1, EKQTLL, interacts with AP1γ1/2 and σ1/2 subunits, but not with AP3 δ and σ (Wang et al., 2014), raising the possibility that these subunits could also be interacting partners of TuMV CI besides AP2β. Although we cannot exclude the possibility that the acidic dileucine motif in CI may be important for binding of other host proteins, our study addresses the function of these motifs in the intracellular targeting of TuMV CI, which is dependent on its interaction with AP2β.

**FIGURE 3:**
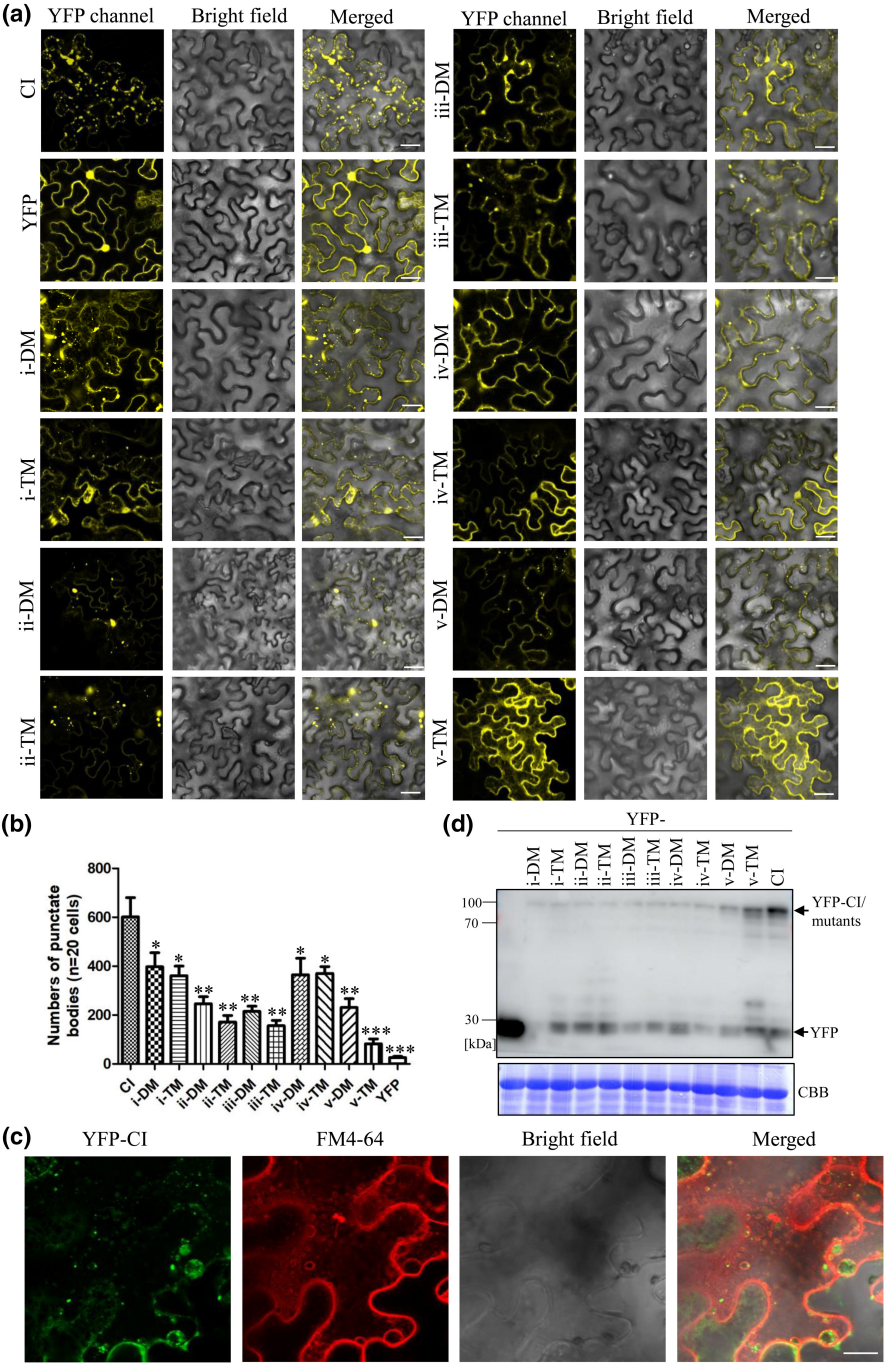
The acidic dileucine motif of cylindrical inclusion (CI) protein is required for endosomal targeting. (a) Subcellular localization of the CI acidic dileucine motif mutants in *Nicotiana benthamiana* leaves. Scale bar = 20 μm. (b) Number of punctate bodies in the cytoplasm when expressing YFP‐CI or its mutants (20 cells per construct were investigated at 2 days post‐agroinfiltration and the numbers were calculated using ImageJ software). Values represent the mean number of punctate bodies ± *SD* per 20 cells from three independent experiments. Statistical significance was determined by Student's *t* test (*p < 0.05, **p < 0.01, ***p < 0.001). (c) Colocalization of FM4‐64 staining with YFP‐CI in *N*. *benthamiana* leaves. Scale bar represents 20 μm. (d) Immunoblotting analysis of YFP‐tagged CI and its mutants in *N*. *benthamiana* leaves using anti‐GFP monoclonal antibody. Coomassie brilliant blue R‐250 (CBB)‐stained RuBisCO large subunit served as a loading control.

Finally, the role of these acidic dileucine motifs of CI in TuMV infection was investigated by alanine mutagenesis of a pCambiaTuMV::GFP infectious clone of TuMV (Cotton et al., 2009). A total of 10 TuMV modified mutants were constructed, designated as TuMV:i‐DM, TuMV:i‐TM, TuMV:ii‐DM, TuMV:ii‐TM, TuMV:iii‐DM, TuMV:iii‐TM, TuMV:iv‐DM, TuMV:iv‐TM, TuMV:v‐DM, and TuMV:v‐TM (Figure 4a). Moreover, a replication‐defective mutant ΔGDD, which has a deletion in the coding sequence for the GDD (glycine‐aspartic acid‐aspartic acid) motif that is the active site of the RNA‐dependent RNA polymerase (Shen et al., 2020), was included. TuMV and its mutants were agroinfiltrated into *N*. *benthamiana* leaves at an OD_600_ of 0.1. At 4 days post‐agroinfiltration (dpai) with wild‐type TuMV there was bright fluorescence in inoculated leaves under UV light (Figure 4b), while the fluorescence was significantly weaker with all the mutants. At 7 dpai, wild‐type TuMV started to induce typical virus symptoms. There were systemic symptoms on all (15/15) plants inoculated with wild‐type TuMV but there was a lower incidence with all the CI mutants and particularly TuMV:ii‐DM, TuMV:ii‐TM, TuMV:iii‐DM, TuMV:iii‐TM, TuMV:v‐DM, and TuMV:v‐TM (20%–40% systemic infection rate), with markedly less fluorescence in the upper noninoculated leaves under UV light (Figure 4c). To determine if these CI mutants compromised viral replication leading to the decreased systemic infection rate, we isolated mesophyll protoplasts from *N*. *benthamiana* leaves and conducted a protoplast transfection assay with wild‐type TuMV, CI mutants, and ΔGDD. The reverse transcription (RT)‐quantitative PCR results showed that the level of accumulation of both positive‐sense and negative‐sense viral RNA resulting from each of the TuMV mutants was significantly less than in the wild type at 40 h after transfection (Figure 4d). The viral replication levels were mostly similar among the CI mutants except that TuMV:v‐TM had significantly less negative‐sense viral RNA accumulation than the other nine (Figure 4d). All CI mutants still had higher replication levels than ΔGDD. The small amount of positive‐sense and negative‐sense viral RNA transcripts of ΔGDD resulted from the activity of the 35S promoter. In *N*. *benthamiana* plants, a western blotting assay to determine the levels of CP accumulation gave consistent results, with lower levels in mutants than in the wild‐type TuMV in both inoculated and upper noninoculated leaves at 4 and 7 dpai (Figure 4e). Notably, six TuMV mutants (TuMV:ii‐DM, TuMV:ii‐TM, TuMV:iii‐DM, TuMV:iii‐TM, TuMV:v‐DM, and TuMV:v‐TM) had substantially reduced CP accumulation compared with wild‐type TuMV and the other mutants (Figure 4e). At 12 dpai, all TuMV infectious clones harbouring CI mutants showed apparently mild symptoms compared with wild‐type TuMV, especially for those six TuMV mutants (TuMV:ii‐DM, TuMV:ii‐TM, TuMV:iii‐DM, TuMV:iii‐TM, TuMV:v‐DM, and TuMV:v‐TM) (Figure S4). ΔGDD showed no symptoms or viral systemic infection. We also verified the sequences of the introduced mutations in progeny viruses recovered from the upper noninoculated leaves by RT‐PCR with primers amplifying the complete CI cistron. The TuMV mutants retained the introduced mutations and no other mutations were observed in them (data not shown). These results suggest that the acidic dileucine motifs identified are involved in viral multiplication.

**FIGURE 4:**
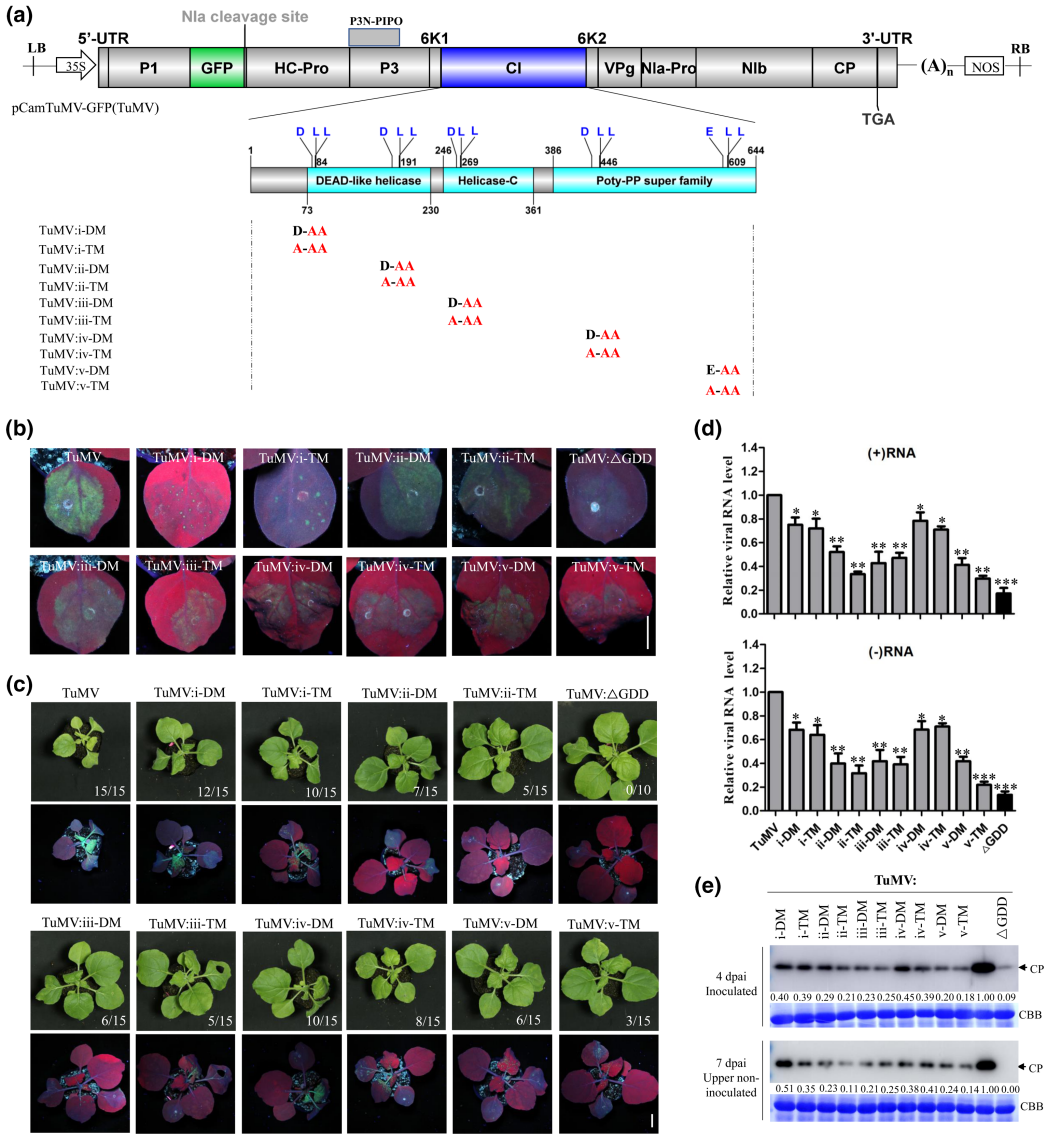
The effect of alanine substitution mutations in the acidic dileucine motif of cylindrical inclusion (CI) protein on the infectivity of TuMV clones in *Nicotiana benthamiana*. (a) Schematic representation of the wild‐type TuMV infectious clone and its mutants harbouring alanine substitutions in the acidic dileucine motifs of the CI protein. (b) Representative photographs of inoculated leaves agroinfiltrated with wild‐type TuMV‐GFP and TuMV mutants taken at 4 days post‐agroinfiltration (dpai) under UV illumination. Scale bar = 1 cm. (c) Representative photographs of *N*. *benthamiana* plants agroinfiltrated with wild‐type TuMV‐GFP (TuMV) and TuMV mutants taken at 7 dpai under normal light (the upper panels) or UV illumination (the lower panels). The fraction numbers represent the number of systemically infected plants/number of inoculated plants. Scale bar = 1 cm. (d) Replication analysis of TuMV infectious clones harbouring CI mutants in protoplasts. Total RNA was extracted from protoplasts of *N*. *benthamiana* leaves transfected with TuMV wild‐type or mutants at 40 h after transfection. Viral (+)‐strand RNA (top panel) and (−)‐strand RNA (bottom panel) were quantified by reverse transcription‐quantitative PCR. Data represent means with *SD* of three biological replicates. Statistical significance was determined by Student's *t* test (*p < 0.05, **p < 0.01, ***p < 0.001). (e) Immunoblotting analysis of TuMV coat protein (CP) accumulation levels of wild‐type TuMV and TuMV mutants in *N*. *benthamiana* leaves using anti‐TuMV CP polyclonal antibody. The relative TuMV CP signals were quantified by ImageJ software. Coomassie brilliant blue R‐250 (CBB)‐stained RuBisCO large subunit served as a loading control.

Genetic evidence suggests that motifs ii, iii, and v play predominant roles in TuMV infection compared with the other two motifs (Figure 4). These three motifs are also much more conserved among potyviruses (Figure 1), suggesting their importance for viral infectivity. Motif v has not been investigated previously, and its triple mutant v‐TM had totally different subcellular localization and much decreased systemic infection (Figures 3, 4, and S4). Moreover, v‐TM had an up to c.2‐fold reduction in TuMV replication compared with v‐DM (Figure 4), suggesting that the single acidic glutamic acid (E) residue plays a predominant role in infection compared with the LL in this motif. Motif iii is also located in the RNA helicase domain of CI (Sorel et al., 2014). Previously, alanine‐scanning mutagenesis of conserved amino acids of TuMV CI revealed that residues at positions 261 and 263, which partially overlap with motif iii in this study, are required for TuMV replication (Deng et al., 2015), suggesting that motif iii might also have an RNA helicase role.

In summary, this work uncovers novel CI functional motifs, as well as molecular mechanisms, underlying the role of the acidic dileucine motif in endosomal targeting of a viral movement protein and viral replication. For a greater understanding of the endosome function in plant viral infection, it will be important to further dissect the roles of other AP complexes.

## AUTHOR CONTRIBUTIONS

F.Y. and J.C supervised this study. G.W. performed most of the experiments, assisted by Z.J., P.R., H.Z., Y.L., L.L., J.P., and S.R. G.W. and F.Y. analysed the data. G.W. and F.Y. wrote the manuscript, assisted by A.W. All authors discussed the results and commented on this manuscript.

## CONFLICT OF INTEREST

The authors declare that they have no competing interests.

## Supporting information


**File S1** Materials and methods

**Figure S1** Bimolecular fluorescence complementation assay of negative controls. GUS‐YN with YC‐fused cylindrical inclusion (CI) mutants or GUS‐YC with AtAP2β‐YN were used as negative controls. Scale bar = 20 μm

**Figure S2** The acidic dileucine motifs in the amino acid sequences of TuMV cylindrical inclusion (CI) protein indicated with red arrows and squares

**Figure S3** Sizes of the punctate structures when cylindrical inclusion (CI) protein and its mutants were expressed in *Nicotiana benthamiana* leaves

**Figure S4** Representative photographs of *Nicotiana benthamiana* plants agroinfiltrated with wild‐type TuMV‐GFP (TuMV), TuMV‐ΔGDD, and TuMV mutants. Photographs were taken at 12 days after agroinfiltration under normal light (the upper panels) or UV illumination (the lower panels). Scale bar = 5 cm

**Table S1** Primers used in this study

## Data Availability

The data that supports the findings of this study are available in the supplementary material of this article.
