# Supplementary material for: Acidic dileucine motifs in the cylindrical inclusion protein of turnip mosaic virus are crucial for endosomal targeting and viral replication
Source: Mol Plant Pathol. 2022 May 25;23(9):1381–9. doi: 10.1111/mpp.13231 (PMC9366067; doi:10.1111/mpp.13231)
Supplement: Supplementary file 1 — File S1 Materials and methods [file MPP-23-1381-s003.docx]

**FILE S1 Materials and methods**

**Plant materials and growth condition**

*N. benthamiana* plants were usually grown in a growth room with a 16 h photoperiod and a relative humidity of 75% at 22 °C. Plants used for protoplast isolation were grown with a 10-h light/14-h dark regime.

**RNA extraction, RT-qPCR, gene cloning and plasmid construction**

Total RNA isolation, the first cDNA synthesis and qPCR were conducted as previously described (Wu *et al.*, 2018). TuMV coding regions were amplified from the TuMV infectious clone (Cotton *et al.*, 2009), using primers listed in **Supplemental Table S1**.

Phusion^®^ High-Fidelity DNA Polymerase (New England Biolabs, USA) was used to amplify all DNA sequences, and Gateway technology (Thermo Fisher Scientific, USA) was employed for plasmid construction. For BiFC assays, the TuMV genes and host gene were transferred into the p35S-gateway-YFP-C and p35S-gateway-YFP-N (Tian *et al.*, 2011). The pBA-FLAG-4×Myc-DC vector (Zhu *et al.*, 2011) was used to yield a FLAG-4×Myc-tagged construct. Mutagenesis was performed using overlapping PCR or the QuickChange II XL site-directed mutagenesis kit (Agilent), according to the manufacturer’s instructions. All constructs were verified by sequencing to ensure that no errors were introduced by PCR amplification.

**Transient expression in *N. benthamiana***

Binary vectors were introduced into *Agrobacterium tumefaciens* GV3101 cells via electroporation. BiFC assays were conducted as previously reported (Tian et al., 2011). For subcellular localization assays, the OD_600_ was adjusted to 0.1. For TuMV agro-infiltration assays, the OD_600_ was adjusted to 0.1-0.2 unless otherwise specified. Three- to four-week-old *N*. *benthamiana* plants were used for agroinfiltration. Co-immunopurification experiments were carried out with anti-flag M2 affinity gel as previously described (Win *et al.*, 2011, Wu *et al.*, 2020). Total protein extraction and immunoblotting was performed as previously described (Wu *et al.*, 2022).

**Confocal microscopy and FM4-64 staining**

*N*. *benthamiana* leaves were infiltrated with 20 μM FM4-64 (Invitrogen) before microscopy and imaging as previously described (Rigal *et al.*, 2015, Wu *et al*., 2020). The agroinfiltrated leaf tissues of *N. benthamiana* leaves were observed at 48 to 72 h post inoculation unless otherwise specified on a Nikon A1R HD25 confocal microscope (Nikon Microsystems, Japan) (Wu et al., 2020). Fluorescence signals for GFP (488 nm/496–518 nm), YFP (514 nm/529–550 nm), and mRFP (543 nm/593–636 nm) were detected. Sequential scanning was used to avoid any interference between fluorescence channels. Image processing was performed with the NIS-elements viewer 4 software (Nikon Microsystem).

**Image J analysis of data**

Image J was used to quantify average integrated density values of bands on immunoblots and punctate bodies in confocal images. The number of punctate structures of YFP in the cytoplasm was calculated under a fluorescence microscope with the same settings. Signals close to perinuclear membranes and big YFP aggregates were excluded. The scanned images were saved in 16bit.tiff format. The relative density of the peaks in selected areas and the punctate bodies in confocal images was calculated by Image J software as described in the manual.

## Protoplast isolation and plasmid transfection

Mesophyll protoplasts were prepared from four-week-old *N. benthamiana* leaves by the procedure described previously (Yoo *et al.*, 2007, Dai *et al.*, 2020). About 1×10^5^ protoplasts were transfected with 10 µg TuMV infectious clone plasmids for viral replication assays (Wu *et al*., 2020, Dai et al., 2020).

**Accession Numbers**

Sequence data from this article can be found in the Arabidopsis Genome database under accession number AT4G23460 (AtAP2β) and in GenBank under accession number EF028235.1 for TuMV.

**References**

Cotton, S., Grangeon, R., Thivierge, K., Mathieu, I., Ide, C., Wei, T.*, et al.* (2009) Turnip mosaic virus RNA replication complex vesicles are mobile, align with microfilaments, and are each derived from a single viral genome. *Journal of Virology,* **83,** 10460-10471.

Dai, Z. J., He, R. R., Bernards, M. A. and Wang, A. M. (2020) The cis-expression of the coat protein of turnip mosaic virus is essential for viral intercellular movement in plants. *Molecular Plant Pathology,* **00,** 1-18.

Rigal, A., Doyle, S. M. and Robert, S. (2015) Live cell imaging of FM4-64, a tool for tracing the endocytic pathways in Arabidopsis root cells. *Methods in Molecular Biology,* **1242,** 93-103.

Tian, G., Lu, Q., Zhang, L., Kohalmi, S. E. and Cui, Y. (2011) Detection of Protein Interactions in Plant using a Gateway Compatible Bimolecular Fluorescence Complementation (BiFC) System. *Journal of Visualized Experiments,* **55,** 1-3.

Win, J., Kamoun, S. and Jones, A. M. E. (2011) Purification of Effector-Target Protein Complexes via Transient Expression in Nicotiana benthamiana. *Methods in Molecular Biology,* **712,** 181-194.

Wu, G., Jia, Z., Ding, K., Zheng, H., Lu, Y., Lin, L.*, et al.* (2022) Turnip mosaic virus co-opts the vacuolar sorting receptor VSR4 to promote viral genome replication in plants by targeting viral replication vesicles to the endosome. *PLoS Pathogens,* **18,** e1010257.

Wu, G. W., Cui, X. Y., Chen, H., Renaud, J. B., Yu, K. F., Chen, X.*, et al.* (2018) Dynamin-like proteins of endocytosis in plants are coopted by Potyviruses to enhance virus Infection. *Journal of Virology,* **92,** e01320-01318.

Wu, G. W., Cui, X. Y., Dai, Z. J., He, R. R., Li, Y. Z., Yu, K. F.*, et al.* (2020) A plant RNA virus hijacks endocytic proteins to establish its infection in plants. *Plant Journal,* **101,** 384-400.

Yoo, S. D., Cho, Y. H. and Sheen, J. (2007) Arabidopsis mesophyll protoplasts: a versatile cell system for transient gene expression analysis. *Nature Protocal,* **2,** 1565-1572.

Zhu, H., Hu, F., Wang, R., Zhou, X., Sze, S. H., Liou, L. W.*, et al.* (2011) Arabidopsis Argonaute10 specifically sequesters miR166/165 to regulate shoot apical meristem development. *Cell,* **145,** 242-256.
