# Supplementary figures and images for: Acidic dileucine motifs in the cylindrical inclusion protein of turnip mosaic virus are crucial for endosomal targeting and viral replication
Source: Mol Plant Pathol. 2022 May 25;23(9):1381–9. doi: 10.1111/mpp.13231 (PMC9366067; doi:10.1111/mpp.13231)

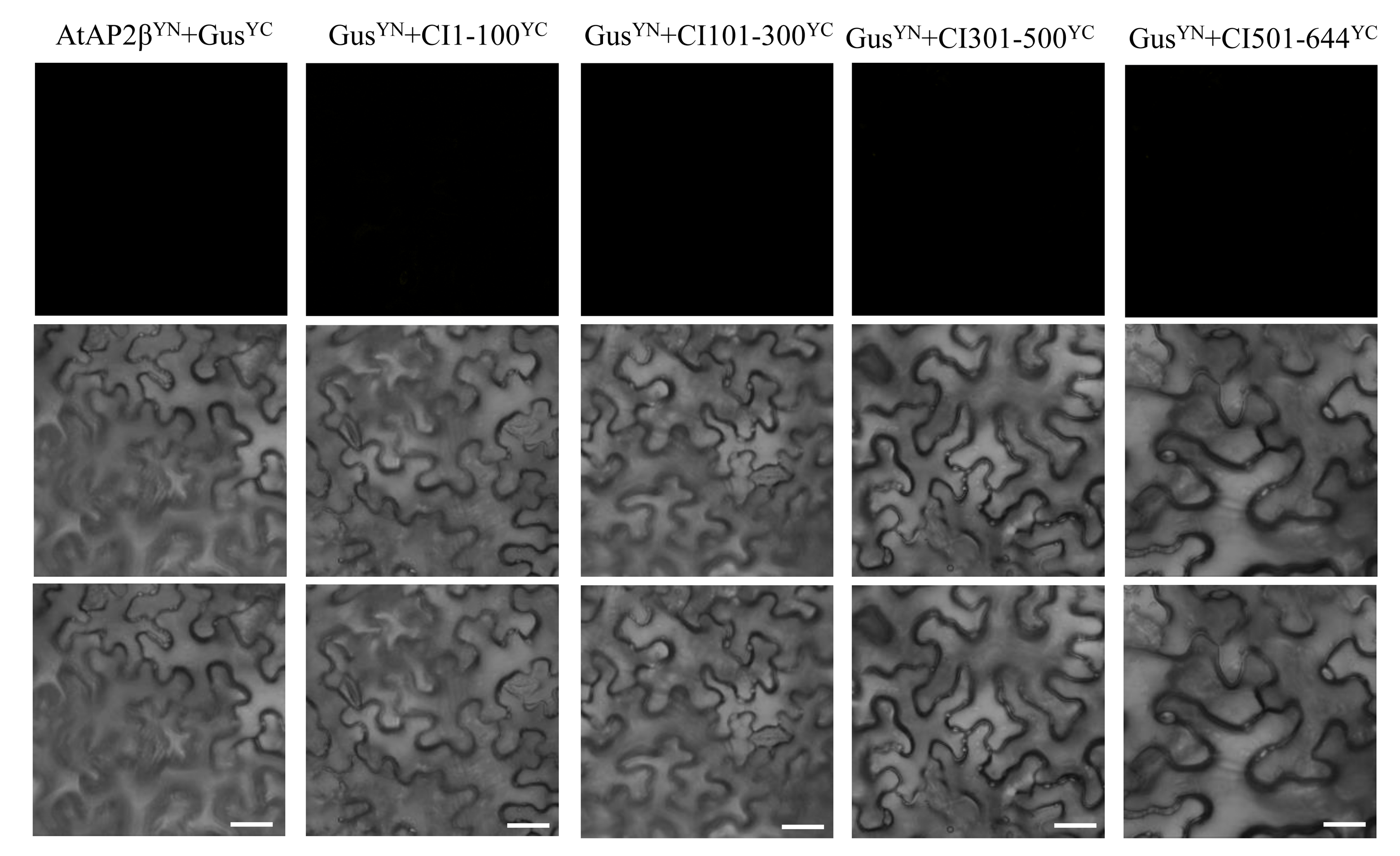

Supplement: Supplementary file 2 — Figure S1 Bimolecular fluorescence complementation assay of negative controls. GUS‐YN with YC‐fused cylindrical inclusion (CI) mutants or GUS‐YC with AtAP2β‐YN were used as negative controls. Scale bar = 20 μm [file MPP-23-1381-s005.tif]

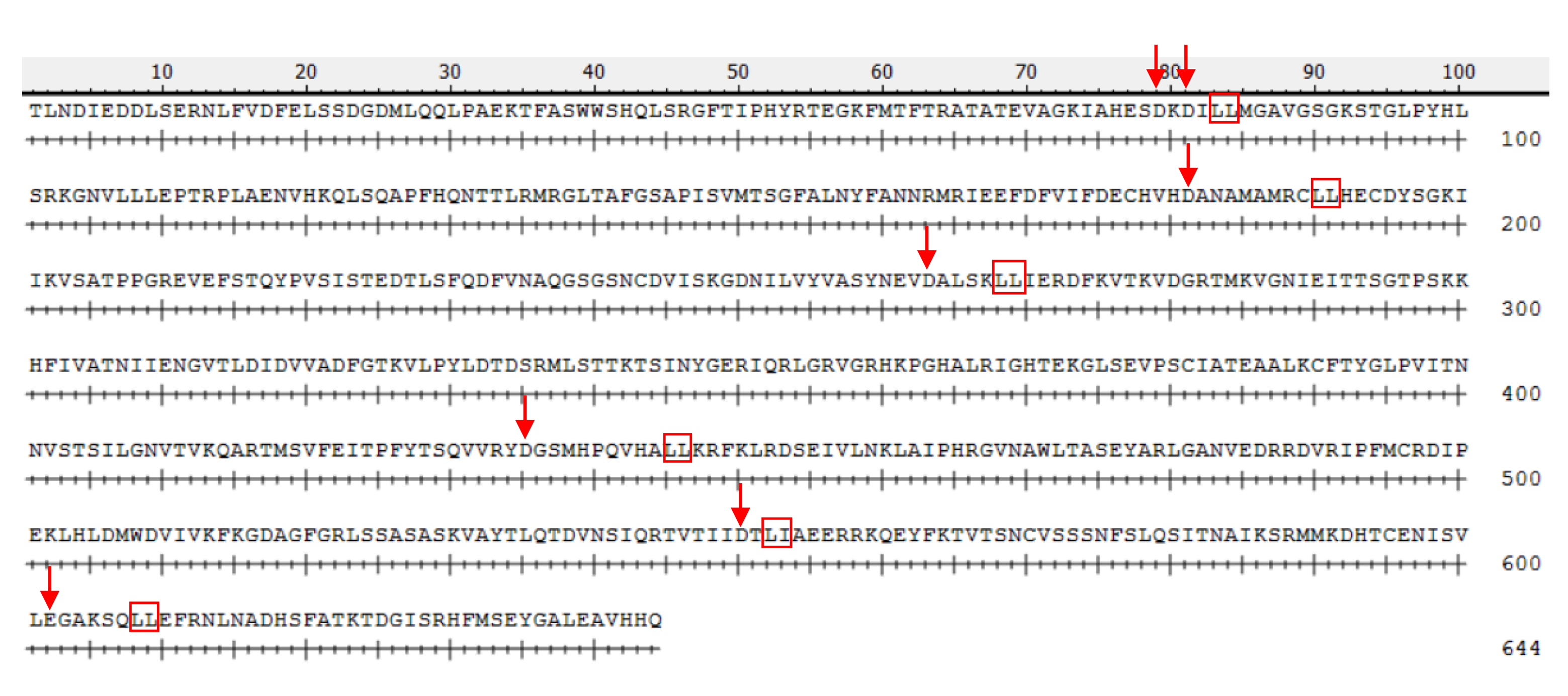

Supplement: Supplementary file 3 — Figure S2 The acidic dileucine motifs in the amino acid sequences of TuMV cylindrical inclusion (CI) protein indicated with red arrows and squares [file MPP-23-1381-s001.tif]

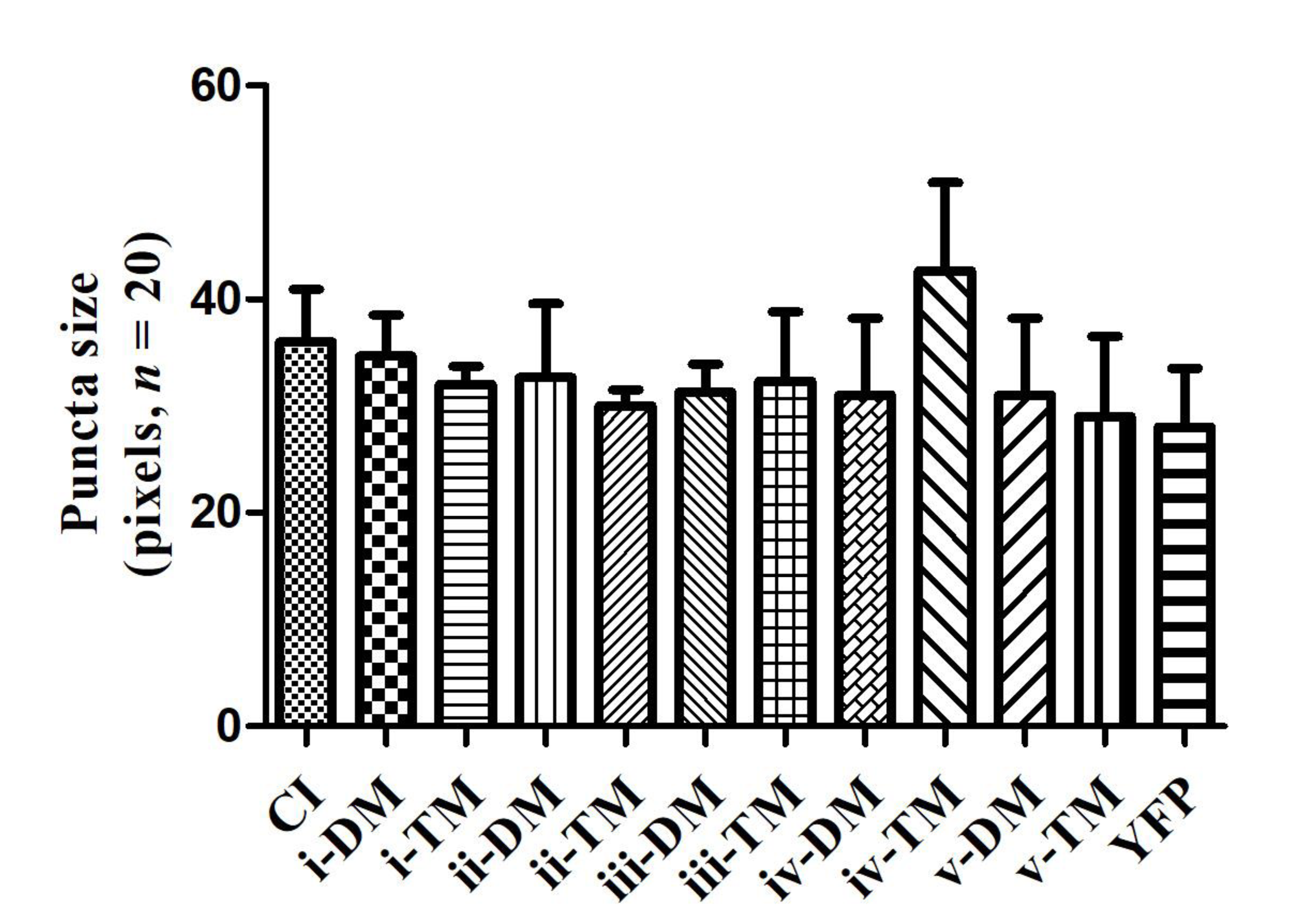

Supplement: Supplementary file 4 — Figure S3 Sizes of the punctate structures when cylindrical inclusion (CI) protein and its mutants were expressed in Nicotiana benthamiana leaves [file MPP-23-1381-s004.tif]

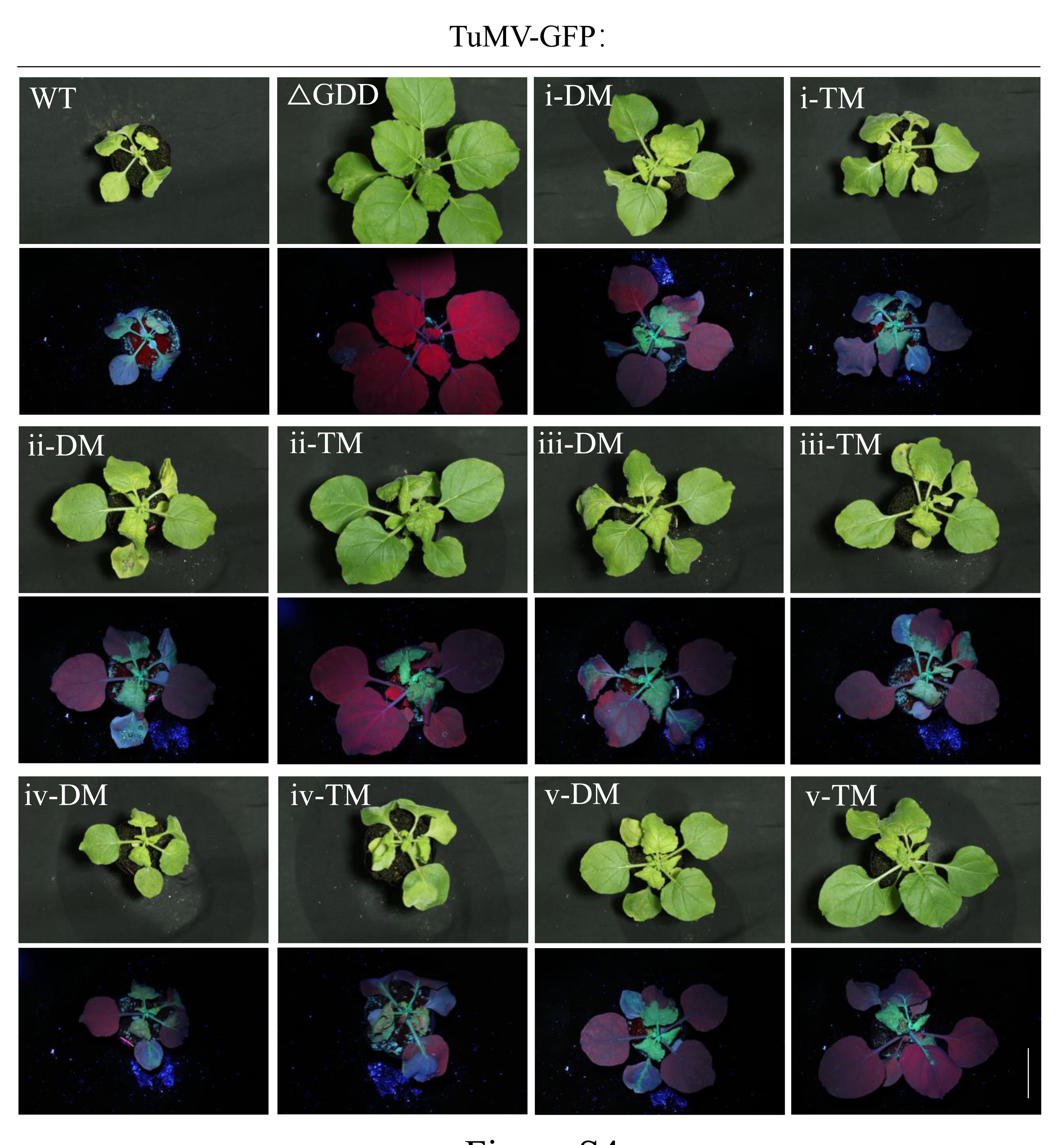

Supplement: Supplementary file 5 — Figure S4 Representative photographs of Nicotiana benthamiana plants agroinfiltrated with wild‐type TuMV‐GFP (TuMV), TuMV‐ΔGDD, and TuMV mutants. Photographs were taken at 12 days after agroinfiltration under normal light (the upper panels) or UV illumination (the lower panels). Scale bar = 5 cm [file MPP-23-1381-s002.tif]
