# Supplementary material for: Acidic dileucine motifs in the cylindrical inclusion protein of turnip mosaic virus are crucial for endosomal targeting and viral replication
Source: Mol Plant Pathol. 2022 May 25;23(9):1381–9. doi: 10.1111/mpp.13231 (PMC9366067; doi:10.1111/mpp.13231)
Supplement: Supplementary file 6 — Table S1 Primers used in this study [file MPP-23-1381-s006.docx]

**Supplemental Table S1.** Primers used in this study.

| **Primer names** | **Sequences (5’ to 3’)** | **Notes** |
| --- | --- | --- |
| BP-TuMVCI-F | GGGGACAAGTTTGTACAAAAAAGCAGGCTTCATGactctcaatgatatagaggatg | Primers are designed for cloning into the entry vector pDONR221.  GenBank Accession no.: NM_127036.5 |
| BP-TuMVCI-R | GGGGACCACTTTGTACAAGAAAGCTGGGTCttgatggtgaactgcctcaag |  |
| BP-TuMVCI1-100-R | GGGGACCACTTTGTACAAGAAAGCTGGGTCgagatgatatggcaagccag | Primers are designed based on the submitted NCBI sequence for the TuMV isolate (EF028235.1). |
| BP-TuMVCI101-300-F | GGGGACAAGTTTGTACAAAAAAGCAGGCTTCATGtccagaaaagggaacgtattac |  |
| BP-TuMVCI101-300-R | GGGGACCACTTTGTACAAGAAAGCTGGGTCcttcttactaggtgttccac |  |
| BP-TuMVCI301-500-F | GGGGACAAGTTTGTACAAAAAAGCAGGCTTCATGcacttcatagttgcaaccaac |  |
| BP-TuMVCI301-500-R | GGGGACCACTTTGTACAAGAAAGCTGGGTCtgggatgtcgcgacacataaaag |  |
| BP-TuMVCI501-644-F | GGGGACAAGTTTGTACAAAAAAGCAGGCTTCATGgaaaaacttcatctagacatg |  |
| CI-D79A-83AA84-F | ggtaaaatagcacacgagagtGCTaaagacataGCAGCGatgggagcagtaggatcag |  |
| CI-D79A-83AA84-R | CGCTGCtatgtctttAGCactctcgtgtgctattttacc |  |
| CI-83AA84-F | cacgagagtgacaaagacataGCAGCGatgggagcagtaggatcaggtaag |  |
| CI-83AA84-R | tcccatCGCTGCtatgtctttgtcactctcgtg |  |
| CI-D190AA191-F | gccaatgcaatggcgatgagatgtGCAGCGcatgagtgtgactattc |  |
| CI-D181AAA191-R | CTGCacatctcatcgccattgcattggcTGCatgaacgtgacattcatc |  |
| CI-190AA191-R | CTGCacatctcatcgccattgcattggcgtcatgaacgtgacattcatc |  |
| CI-D263A-268AA269-F | gtagcaagctacaatgaggtaGCTgcgctttcaaaaGCAGCG attgaaagagacttcaaagtc |  |
| CI-D263A-268AA269-R | CGCTGCttttgaaagcgcAGCtacctcattgtagcttgctac |  |
| CI-268AA269-F | ctacaatgaggtagacgcgctttcaaaaGCAGCGattgaaagagacttcaaagtcacg |  |
| CI-D435A-445AA446-F | gctccatgcatccacaggtgcacgcaGCAGCGaagagattcaaactcag |  |
| CI-D435A-445AA446-R | tgcgtgcacctgtggatgcatggagccTGCatatctcaccacttggct |  |
| CI-445AA446-R | tgcgtgcacctgtggatgcatggagccATCatatctcaccacttggct |  |
| CI-E602A-608AA609-F | cttGCAggagcgaagtcacagGCAGCGgagtttagaaacctgaatg |  |
| CI-E602A-608AA609-R | ctcCGCTGCctgtgacttcgctccTGCaagcactgatatgttctc |  |
| CI-E608AA609-F | gaaggagcgaagtcacagGCAGCGgagtttagaaacctgaatg |  |
| CI-E608AA609-R | cCGCTGCctgtgacttcgctccTTCaagcactgatatgttctc |  |
| qPCR-TuMV cp-F | TGGCTGATTACGAACTGACG | Primers are designed based on the submitted NCBI sequence for the TuMV isolate (EF028235.1). |
| qPCR-TuMV cp-R | CTGCCTAAATGTGGGTTTGG |  |
| qPCR-Nbactin-F | GGGATGTGAAGGAGAAGTTGGC | Primers are designed based on the reference sequences (AY179605 for *NbACT* deposited in GenBank). |
| qPCR- Nbactin-R | ATCAGCAATGCCCGGGAACA |  |
